# The effect of pilocarpine on dental caries in patients with primary Sjögren’s syndrome: a database prospective cohort study

**DOI:** 10.1186/s13075-019-2031-7

**Published:** 2019-11-27

**Authors:** Chung-Yuan Hsu, Kuo-Chun Hung, Ming-Shyan Lin, Chi-Hua Ko, Yu-Sheng Lin, Tien-Hsing Chen, Chun-Yu Lin, Ying-Chou Chen

**Affiliations:** 1 Division of Rheumatology, Allergy, and Immunology, Department of Internal Medicine, Kaohsiung Chang Gung Memorial Hospital and Chang Gung University College of Medicine, No. 123, Dapi Rd., Niaosong District, Kaohsiung City, 833 Taiwan; 2 Department of Cardiology, Chang Gung Memorial Hospital, Linkuo Branch, Chang Gung University College of Medicine, Taoyuan, Taiwan; 3 Division of Cardiology, Chang Gung Memorial Hospital, Yunlin, Taiwan; 4 Division of Cardiology, Chang Gung Memorial Hospital, Chiayi, Taiwan; 5 School of Traditional Chinese Medicine, College of Medicine, Chang Gung University, Taoyuan County, Taiwan; 6 Division of Cardiology, Department of Internal Medicine, Chang Gung Memorial Hospital, Chang Gung University College of Medicine, Keelung, Taiwan; 7 Department of Internal Medicine, National Cheng Kung University Hospital, College of Medicine, National Cheng Kung University, No.138, Sheng Li Road, Tainan, 704 Taiwan

**Keywords:** Primary Sjögren’s syndrome, Pilocarpine, Dental caries

## Abstract

**Background:**

Primary Sjögren’s syndrome (pSS) is associated with dental caries. Pilocarpine, a salivary stimulant, can improve the amount and flow rate of saliva in patients with pSS. This study aimed to assess whether the risk of dental caries decreases with the use of pilocarpine in patients with pSS.

**Methods:**

For this prospective cohort study, we identified pSS patients from the catastrophic illnesses registry of the National Health Insurance Research Database of Taiwan between 2009 and 2013. We divided participants into pilocarpine and non-user groups based on the pilocarpine prescriptions available during the first 3-month follow-up. The primary endpoint was dental caries. The secondary endpoints were periodontitis and oral candidiasis. We compared the risk of these oral manifestations using the Cox proportional hazard model.

**Results:**

A total of 4973 patients with new-onset pSS were eligible for analysis. After propensity score matching, we included 1014 patients in the pilocarpine group and 2028 patients in the non-user group. During the mean follow-up of 2.6 years, the number of events was 487 in the pilocarpine group (48.0%) and 1047 in the non-user group (51.6%); however, the difference was not significant (hazard ratio [HR] 0.93, 95% confidence interval [CI] 0.82 to 1.06). Furthermore, there was no significant difference between groups regarding risk of periodontitis (HR 0.91, 95% CI 0.81 to 1.03) and oral candidiasis (HR 1.16, 95% CI 0.70 to 1.94).

**Conclusion:**

Pilocarpine may have no protective effect on dental caries, periodontitis, or oral candidiasis in patients with pSS.

## Introduction

Primary Sjögren’s syndrome (pSS) is one of the most common autoimmune diseases, mainly involving the body’s exocrine glands, especially the salivary glands. It can lead to a decrease in salivary flow rate and xerostomia. Changes in the oral environment lead to an accumulation of oral pathogens, which subsequently causes dental caries and oral candidiasis [1]. Despite regular oral healthcare practices, pSS patients experience more dental caries than healthy subjects and are subjected to radical dental treatments [2]. Poor oral health is associated with high mortality, although some unidentified confounding factors may also exist [3].

Salivary stimulants, such as pilocarpine, are parasympathomimetic drugs that bind to the muscarinic receptors on the exocrine glands and stimulate their secretion. Theoretically, endogenous saliva is better than the saliva supplements traditionally used for preventing dental caries because it contains antimicrobial agents, such as secretory IgA and lysozyme [4]. Therefore, based on expert opinions, the Sjögren’s Syndrome Foundation recommends that, in order to prevent caries, saliva output should be increased by stimulating saliva secretion in pSS patients with the dry mouth [5].

In an animal study, pilocarpine appears to exert the greatest caries-protective effect in partially desalivated rats [6]. However, there is no clinical trial demonstrating that salivary stimulants are linked directly to the prevention of caries or a reduction in existing caries in patients with salivary dysfunction. As the guideline recommends the use of salivary stimulants, there should be at least one human study to confirm this effect. Therefore, we designed this study to investigate whether pilocarpine can prevent dental caries and other oral manifestations in patients with pSS.

## Methods

### Data source

The National Health Insurance program was established by the Taiwanese government in 1995. It currently covers 23 million enrolees, which is more than 99% of the Taiwanese population. We selected patients with pSS from the National Health Insurance Research Database (NHIRD) of Taiwan by determining whether they had a catastrophic disease certificate with pSS [7]. If a specialist diagnoses a patient as having pSS (International Classification of Diseases (ICD)-9: 7102), a condition classified as a catastrophic illness by the Ministry of Health and Welfare of Taiwan, they can apply for a catastrophic illness certificate for the patient. In order to apply for a certificate, the diagnosis must be based on the 1993 classification criteria established by the European Community Study Group [8]. Furthermore, a comprehensive application document and relevant information, including a detailed medical record, laboratory data, imaging report, and pathological study are required to be reviewed by an anonymous rheumatology expert assigned by the NHI Administration. If a patient passes a strict review and obtains a certificate, they are no longer required to make the co-payment for the treatment of the SS-related condition. Patients having a catastrophic illness certificate for pSS were defined as a SS case. The definition of SS has been widely reported in previous NHIRD studies [9–11]. The present study was approved by the Chang Gung Medical Foundation Institutional Review Board (No. 201801950B1).

### Identification of study patients

Patients with SS were identified in the NHIRD registry of patients with catastrophic illnesses between 2009 and 2013. The date of approval of the catastrophic illness certificate was assigned as the index date. The exclusion criteria were as follows: patients aged below 20 years at pSS diagnosis, those with catastrophic illness certificates for other autoimmune diseases (systemic lupus erythematosus, systemic sclerosis, dermatomyositis, polymyositis, rheumatoid arthritis, and vasculitis), those with a diagnosis of SS before 2009 (which can be tracked to 1997), or those with a history of malignancy, hepatitis C, acquired immune deficiency syndrome, sarcoidosis, amyloidosis, or organ transplantation.

**Fig. 1:**
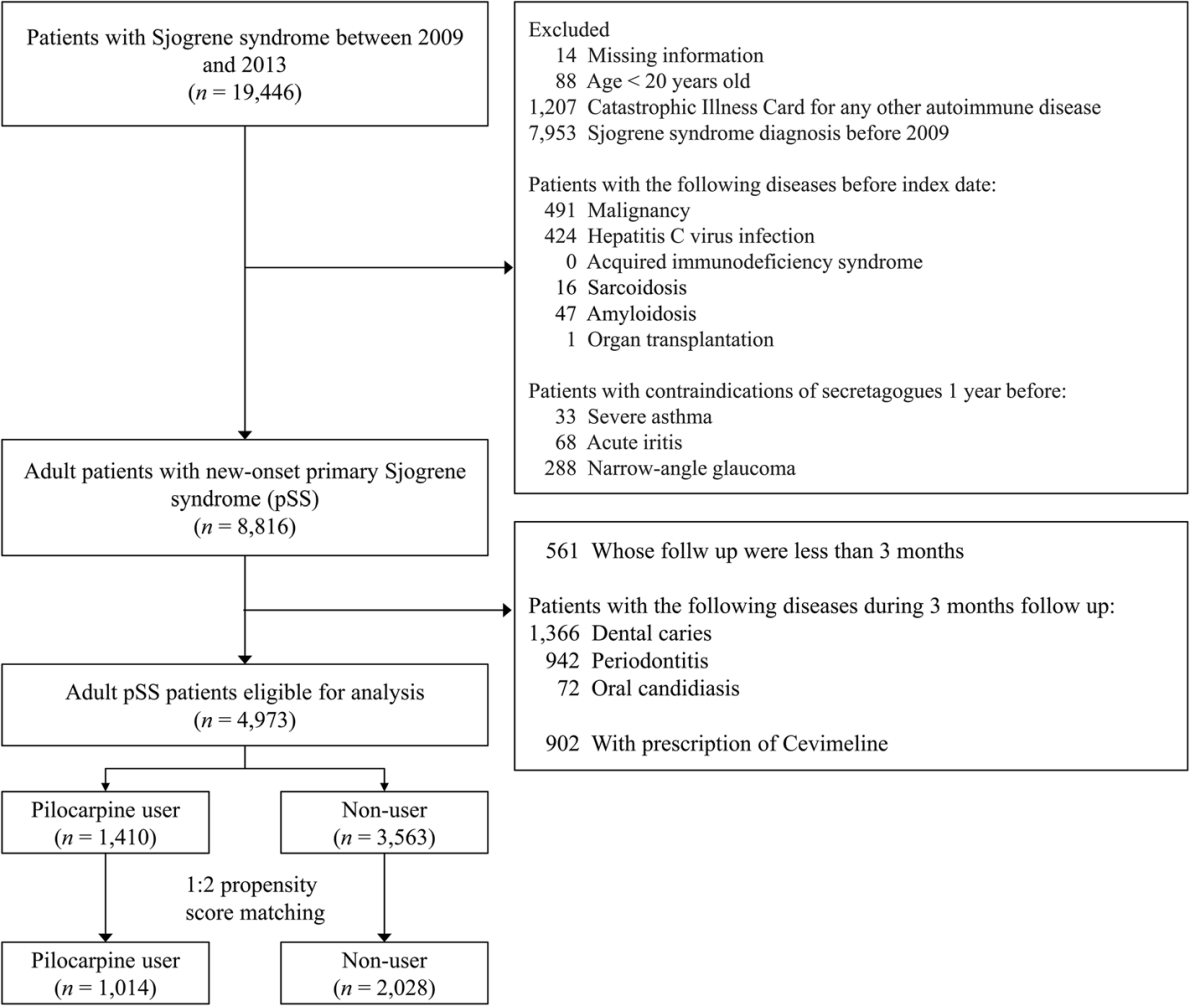
Enrolment of the study patients

Patients with contraindications for salivary stimulants, such as severe asthma, acute iritis, and narrow-angle glaucoma, were excluded. Furthermore, patients with a follow-up of less than 3 months were excluded because the use of salivary stimulants was identified during the first 3-month follow-up starting from the index date. Patients who suffered from an episode of dental caries, periodontitis, or oral candidiasis during the first 3-month follow-up were also excluded. Finally, we excluded patients who were prescribed with cevimeline between 1997 and 2013 because the primary interest of this study was pilocarpine. A total of 4973 patients with new-onset pSS were eligible for final analysis. Of these, 1410 (28.4%) were pilocarpine users and 3563 (71.6%) were non-users (Fig. 1).

### Outcomes

We detected the time-to-event outcomes according to the International Classification of Diseases, Ninth Revision, Clinical Modification Codes (Additional file 1: Table S1). The primary outcome was dental caries, defined as a clinic visit due to tooth decay and receiving the necessary interventions. The event of dental caries was detected using diagnosis codes along with the relevant dental interventions at the dental outpatient visit. Secondary outcomes were the events related to other oral manifestations of pSS including periodontitis, oral candidiasis, and annual number of dentist visits for dental caries and periodontitis. The definition of secondary outcomes has also been frequently adopted in previous studies [12, 13]. The follow-up period was based on the duration between the index date of pSS diagnosis and the onset date of dental caries, date of death, or 31 December 2013, whichever came first.

### Covariate measurement

Comorbidities included diabetes mellitus, chronic kidney disease, cirrhosis, chronic obstructive pulmonary disease, stroke, and dementia. The detection of comorbidities was defined as having at least two outpatient diagnoses or any one diagnosis at admission of the comorbidities during the year before the index date. (Additional file 1: Table S1). Medications prescribed during the first 3-month follow-up including systemic glucocorticoids, azathioprine, cyclophosphamide, hydroxychloroquine (HCQ), methotrexate, and pulse steroid therapy were identified from the prescriptions of the outpatient departments. Cumulative glucocorticoid doses (equivalent to prednisolone) during the first 3-month follow-up were also calculated. The use of potent immunosuppression was also documented, and it was defined as azathioprine, cyclophosphamide, methotrexate, or prednisolone equivalent glucocorticoid dose ≥ 1350 mg (15 mg per day for 90 days). Information on pSS-related healthcare during the first 3-month follow-up, such as the number of emergency room visits, number of admissions, and hospitalisation days, was retrieved.

### Statistical analysis

Propensity score matching was conducted to compare the pilocarpine and non-user groups. We matched each patient from the pilocarpine group with two corresponding counterparts from the non-user group [14]. The propensity score was the predicted probability of being in the pilocarpine group, derived from the value of covariates obtained using multivariable logistic regression analysis. The covariates included to calculate the propensity score were age at diagnosis of pSS, age at the index date, sex, comorbidities (6 items), immunosuppressants (7 items), healthcare utilisation due to pSS (3 items), and index date. Matching was conducted using a greedy nearest neighbour algorithm with a calliper 0.2 times the standard deviation of the logit of propensity score, without replacement and with random matching order. The quality of the matching was checked using the absolute value of standardised mean difference (SMD), in which a value less than 0.1 was considered to be the negligible difference between the two groups [15].

The risk of time-to-event outcomes (dental caries and oral candidiasis) between the two groups was compared using the Cox proportional hazard model. The matching pairs were stratified in the Cox model. The annual number of dentist visits for dental caries or periodontitis in the two groups was compared using the Poisson log-linear model, in which the logarithm of the follow-up duration was set as an offset variable. The within-cluster correlation of the same matching pair was considered by introducing the work correlation matrix of the generalised estimating equation (GEE). The study group (pilocarpine versus non-user) was the only explanatory variable in the regression analyses. Finally, several subgroup analyses of dental caries were performed using the pre-specified subgroups of age, sex, and HCQ use. A two-sided *p* value of < 0.05 was considered statistically significant. No adjustment for multiple testing (multiplicity) was made in this study. All statistical analyses, including the ‘*psmatch*’ procedures for propensity score matching, ‘*genmod*’ for GEE, and ‘*phreg*’ for survival analyses, were performed using commercial software (SAS 9.4, SAS Institute, Cary, NC).

## Results

### Patient characteristics

The distributions of patients’ characteristics in the pilocarpine and non-user groups before and after propensity score matching are presented in Table 1. Before matching (left panel in Table 1), patients in the pilocarpine group were older; were more likely to be prescribed HCQ; were less likely to be prescribed corticosteroid, azathioprine, or pulse steroid; and had a lower number of hospital admissions and hospitalisation days due to pSS during follow-up. Mean follow-up duration was 2.3 and 2.5 years in the pilocarpine and non-user groups, respectively. After matching, the distribution of patients’ characteristics in the two groups was homogeneous and comparable with the absolute values of SMD (< 0.1) (right panel in Table 1).

**Table 1:** Characteristics of the study patients before and after propensity score matching

	Before matching			After matching		
Variable	Pilocarpine (*n* = 1410)	Non-user (*n* = 3563)	SMD	Pilocarpine (*n* = 1014)	Non-user (*n* = 2028)	SMD
Characteristic						
Age at diagnosis of newly onset pSS (years)	57.0 ± 13.5	52.9 ± 14.6	0.296	54.7 ± 13.3	54.2 ± 13.5	0.034
Age group						
≤ 40 years	156 (11.1)	722 (20.3)	− 0.255	146 (14.4)	288 (14.2)	0.006
41–65 years	861 (61.1)	2118 (59.4)	0.033	644 (63.5)	1324 (65.3)	− 0.037
> 65 years	393 (27.9)	723 (20.3)	0.178	224 (22.1)	416 (20.5)	0.039
Female gender	1271 (90.1)	3183 (89.3)	0.027	915 (90.2)	1811 (89.3)	0.031
Comorbidity in the previous year						
Diabetes mellitus	146 (10.4)	285 (8.0)	0.082	83 (8.2)	163 (8.0)	0.005
Chronic kidney disease	78 (5.5)	180 (5.1)	0.021	46 (4.5)	86 (4.2)	0.014
Cirrhosis	9 (0.6)	47 (1.3)	− 0.069	8 (0.8)	11 (0.5)	0.030
Chronic obstructive pulmonary disease	64 (4.5)	151 (4.2)	0.015	47 (4.6)	83 (4.1)	0.027
Stroke	65 (4.6)	134 (3.8)	0.042	36 (3.6)	74 (3.6)	− 0.005
Dementia	10 (0.7)	42 (1.2)	− 0.049	8 (0.8)	20 (1.0)	− 0.021
Immunosuppressant during the 3 months of follow-up						
Prednisolone equivalent glucocorticoid dose of glucocorticoid users	142.8 ± 307.3	280.2 ± 785.1	− 0.230	157.1 ± 346.4	176.3 ± 385.7	− 0.052
Systemic glucocorticoids	533 (37.8)	1501 (42.1)	− 0.088	406 (40.0)	777 (38.3)	0.035
Azathioprine	42 (3.0)	244 (6.8)	− 0.180	41 (4.0)	74 (3.6)	0.021
Cyclophosphamide	6 (0.4)	42 (1.2)	− 0.085	5 (0.5)	7 (0.3)	0.023
Hydroxychloroquine	1228 (87.1)	2871 (80.6)	0.178	859 (84.7)	1737 (85.7)	− 0.026
Methotrexate	38 (2.7)	133 (3.7)	− 0.059	32 (3.2)	61 (3.0)	0.009
Pulse steroid therapy	2 (0.1)	36 (1.0)	− 0.115	2 (0.2)	8 (0.4)	− 0.036
Potent immunosuppression	87 (6.2)	438 (12.3)	− 0.213	79 (7.8)	149 (7.3)	0.017
Healthcare utilisation due to pSS during the follow-up						
Number of ER visits per 5 years	0.57 ± 2.12	0.76 ± 4.04	− 0.057	0.55 ± 1.93	0.64 ± 3.27	− 0.036
Number of admissions per 5 years	0.34 ± 1.59	0.67 ± 2.81	− 0.145	0.42 ± 1.78	0.46 ± 2.31	− 0.022
Hospitalisation days per 5 years	2.64 ± 17.02	6.45 ± 36.20	− 0.134	3.40 ± 19.71	3.82 ± 24.95	− 0.019
Follow-up (years)	2.3 ± 1.5	2.5 ± 1.3	−0.189	2.7 ± 1.3	2.6 ± 1.3	0.011
Propensity score	0.335 ± 0.122	0.263 ± 0.114	0.608	0.277 ± 0.079	0.274 ± 0.079	0.030

*SMD* standardised mean difference, *pSS* primary Sjögren’s syndrome, *ER* emergency room

### Primary and secondary outcomes

**Fig. 2:**
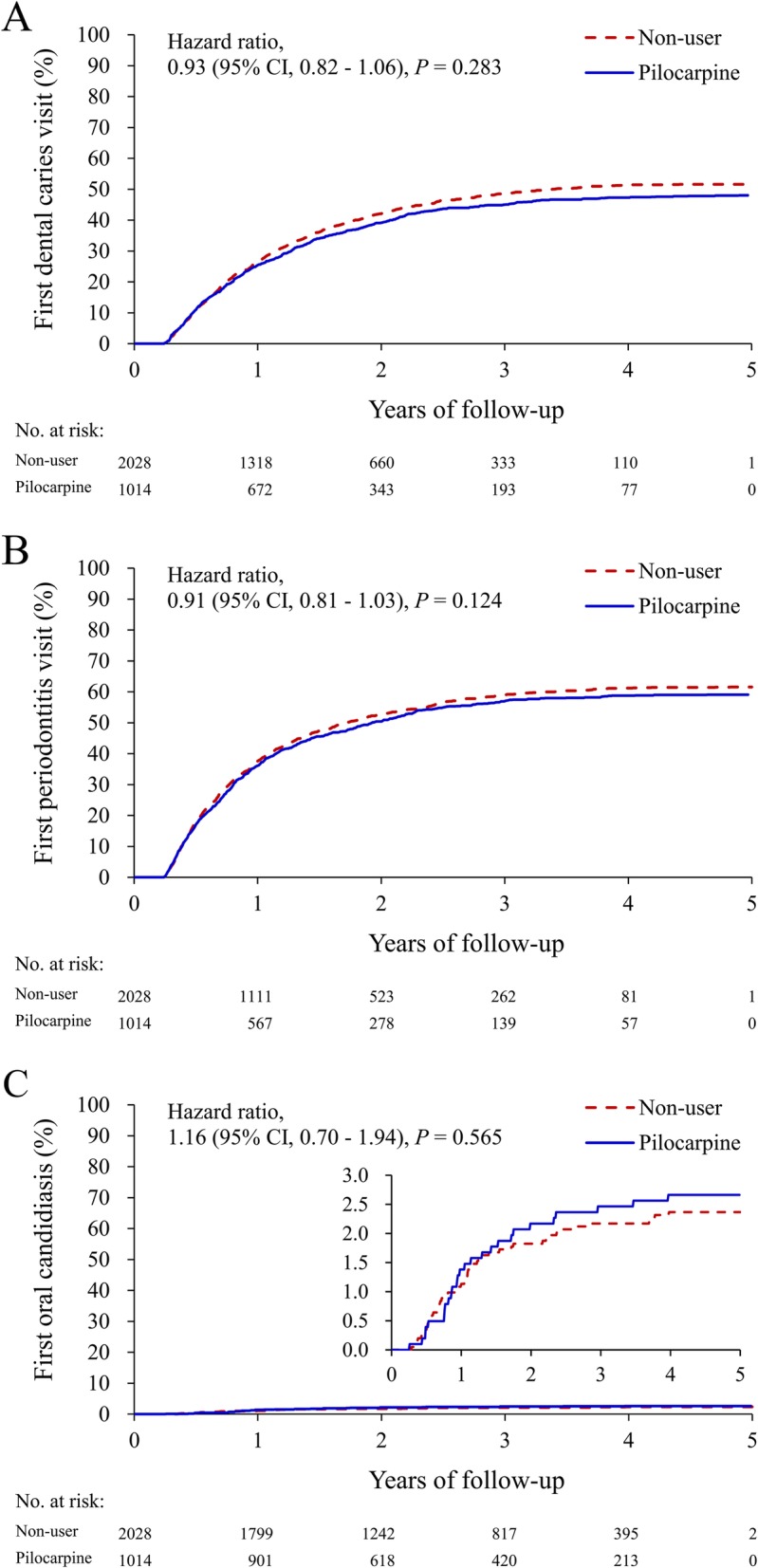
Unadjusted cumulative event rates of dental caries (**a**), periodontitis (**b**), and oral candidiasis (**c**) in pSS patients with or without pilocarpine

During the mean follow-up of 2.6 years (standard deviation 1.3 years), there were 487 (48.0%) and 1047 (51.6%) patients with dental caries in the pilocarpine and non-user groups, respectively (Table 2). The cumulative event rates for dental caries, periodontitis, and oral candidiasis, during follow-up, are shown in Fig. 2a–c. The results indicated that the risk of dental caries was not significantly different between the two groups (hazard ratio [HR] 0.93, 95% confidence interval [CI] 0.82 to 1.06). In addition, there was no significant difference between the groups regarding annual number of dentist visits for dental caries (rate ratio 0.95, 95% CI 0.85 to 1.07), risk of periodontitis (HR 0.91, 95% CI 0.81 to 1.03), annual number of dentist visits for periodontitis (rate ratio 1.004, 95% CI 0.90 to 1.11), and risk of oral candidiasis (HR 1.16, 95% CI 0.70 to 1.94).

**Table 2:** Oral manifestations and mortality during the follow-up period

Variable	Pilocarpine (*n* = 1014)	Non-user (*n* = 2028)	*p*
First dental caries visit			
Event number, *n* (%)	487 (48.0)	1047 (51.6)	
Incidence density (95% CI)^§^	27.4 (25.0–29.9)	30.5 (28.7–32.4)	
Hazard ratio (95% CI)	0.93 (0.82, 1.06)	Reference	0.283
Annual number of dentist visiting for dental caries			
Annual number (mean ± SD)	0.70 ± 1.11	0.75 ± 1.18	
Rate ratio (95% CI)	0.95 (0.85, 1.07)	Reference	0.433
First periodontitis visit			
Event number, *n* (%)	599 (59.1)	1248 (61.5)	
Incidence density (95% CI)^§^	39.1 (35.9–42.2)	41.8 (39.5–44.1)	
Hazard ratio (95% CI)	0.91 (0.81, 1.03)	Reference	0.124
Annual number of dentist visiting for periodontitis			
Annual number (mean ± SD)	0.78 ± 1.06	0.80 ± 1.07	
Rate ratio (95% CI)	1.004 (0.90, 1.11)	Reference	0.942
First oral candidiasis			
Event number, *n* (%)	27 (2.7)	48 (2.4)	
Incidence density (95% CI)^§^	1.02 (0.64–1.40)	0.91 (0.65–1.17)	
Hazard ratio (95% CI)	1.16 (0.70, 1.94)	Reference	0.565

*CI* confidence interval, *SD* standard deviation;

^§^Incidence density: number of events per 100 person-years

### Subgroup analyses

**Fig. 3:**
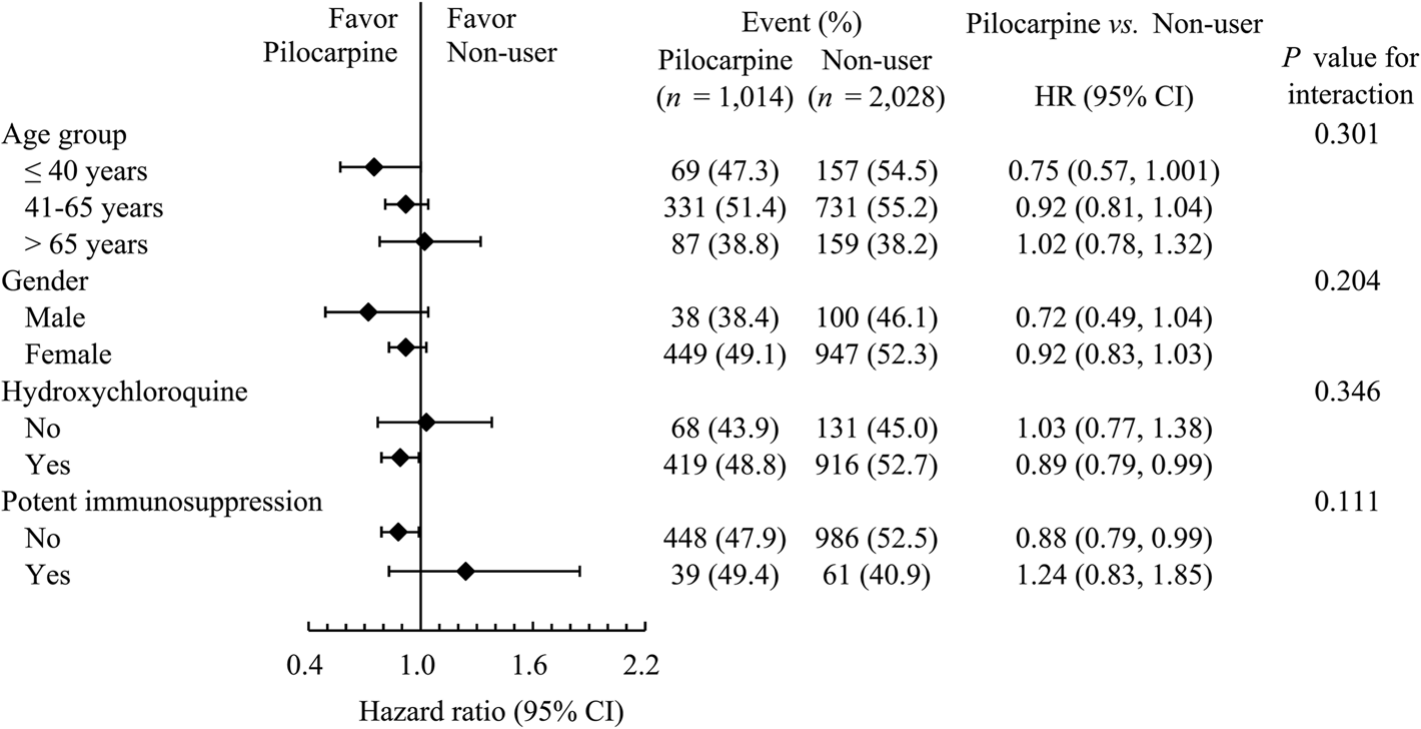
Pre-specified subgroup analysis of dental caries

Subgroup analyses of the primary outcome were further performed using the following pre-defined subgroups: age, sex, HCQ use, and potent immunosuppression (Fig. 3). The results showed that the observed neutral effect of pilocarpine was consistent among different levels of these subgroups. In addition, the protective effect of pilocarpine against dental caries was observed in patients taking HCQ (HR 0.89; 95% CI 0.79 to 0.99); however, the interaction effect was not significant (*p* = 0.346). Moreover, we observed the protective effect of pilocarpine against dental caries in patients not taking potent immunosuppression (HR 0.89; 95% CI 0.79 to 0.99), but the interaction effect was not significant (*p* = 0.111) at the threshold of *p* < 0.05.

In the pilocarpine group, 72.8% of patients had a medication possession ratio (MPR) ≥ 50%. To determine whether higher adherence to pilocarpine resulted in better outcomes, we further subdivided the patients into three groups (non-user, MPR < 50%, and MPR ≥ 50%) according to MPR of pilocarpine during the first 3 months. Further subgroup analysis of the primary outcome was performed according to these adherence subgroups. There was no significance in the trend test indicating that a higher adherence was not associated with a decreased risk of dental caries (*p* = 0.169; Additional file 2: Figure S1).

## Discussion

According to the clinical practice guidelines, salivary stimulation is recommended for caries prevention in pSS patients with dry mouth, and this approach is widely accepted, as indicated by strong anecdotal evidence and clinical experience. Although the data from animal models suggest a possible benefit of pilocarpine, regarding dental caries, the efficacy of oral pilocarpine therapy for dental caries prophylaxis or prevention of oral infections in humans is not known. To the best of our knowledge, this is the first large-scale nationwide population-based study to examine the relationship between the use of pilocarpine and the risk of dental caries in patients with pSS. However, the results of the present study showed that pilocarpine has a neutral effect on dental caries in patients with pSS.

According to the acidogenic theory describing the cause of dental caries, four important factors are required for caries formation: susceptible tooth surface, caries-causing bacteria, fermentable carbohydrates, and time. Saliva is one of the modifying factors that adjust the process [16]. A reduction in salivary flow rate is associated with increased risk for dental caries because the buffering capability of saliva is inadequate to offset the acidic environment created by certain foods [17]. Despite having excellent oral hygiene, pSS patients with dry mouth have a high rate of dental caries and tooth loss even in the early phase. Saliva stimulants, such as pilocarpine, can increase the secretion of saliva, which can theoretically improve tooth decay. Our study results seem inconsistent with this theoretical idea. There may be two explanations for this result, which are discussed in the following two sections.

A randomised control trial has shown that administration of pilocarpine is well tolerated and significantly improves salivary flow and dry mouth symptoms in patients with SS [18]. However, 61% of patients had global improvement of dry mouth, so not every patient with pSS responds well to pilocarpine. Such non-responsiveness can be associated with certain risk factors, such as higher grades of scintigraphic patterns, more lymphocytic infiltrates in salivary gland biopsies, and longer disease duration [19]. Moreover, this may be due to a delayed diagnosis, which is a common and substantial problem, suggesting unmet health needs for pSS [20]. Although some non-responsiveness may lead to insignificant protection, we do not know if patients who respond to pilocarpine will be protected against dental caries. The main limitation of NHIRD is the lack of data on patients’ saliva flow rate, which may introduce bias in the analysis. We cannot rule out the possibility that the same analysis performed only on responder patients may produce different results, such as increasing the secretion of saliva can theoretically improve tooth decay. Not only the flow of saliva but also its composition can increase the susceptibility to dental caries in patients with pSS. The saliva of pSS patients has a low buffering capacity and pH [21]. Furthermore, pSS patients may have high salivary β2-microglobulin, Na^+^, and Cl^−^ levels, which are useful markers for differentiating pSS patients from healthy controls [22]. There is currently no evidence suggesting that salivary stimulants can correct the abnormality in saliva composition. Therefore, the theory of using pilocarpine to prevent tooth decay is doubtful.

In the current study, we addressed other oral manifestations, such as periodontitis and oral candidiasis, as secondary endpoints. No significant decrease in risk was found even in the pilocarpine group. These manifestations are considered to be induced mainly by a decreased clearance in the oral cavity because of an inadequate amount of saliva. Besides, deterioration in saliva quality or loss of the beneficial physiological effects of saliva on the intraoral environment is believed to be another causative factor for refractory intraoral manifestations in pSS [23]. Although a previous study showed that stimulation of salivary flow results in a decrease in Candida colony-forming units, it is still not proven whether a reduced number of colonies in the oral cavity can prevent the onset of oral candidiasis [24].

Therapies involving proper immunomodulation are important because pSS is a disorder of the immune system. However, no significant difference has been observed between HCQ and placebo in treating the symptoms of dryness [25, 26]. Recent data have shown that HCQ can decrease the incidences of extra-glandular manifestations in pSS patients [27]; therefore, it is mainly prescribed for such manifestations. Furthermore, dryness is associated with the presence of several constitutional symptoms, such as fatigue and pain [28]. Based on the evidence, we deduced that patients use HCQ because of the presence of constitutional symptoms, which may lead to more dryness in the mouth. Overall, the current study results showed that the incidence of dental caries was higher in patients taking HCQ. Interestingly, the protective effect of pilocarpine against dental caries was only observed in the subgroup of patients taking HCQ. These patients might have more dryness in the mouth and are more likely to benefit from pilocarpine. Therefore, further trials are needed to confirm whether the effect of pilocarpine differs between special pSS subgroups. On the other hand, subgroup analyses of different pilocarpine adherence did not show a protective effect, even in patients with better adherence. Pilocarpine has no dose-dependent effect on dental caries in pSS patients.

In previous studies, even patients with extra-glandular manifestations did not have dry symptoms, but their risk of developing dental caries was still significantly increased [29]. In the current study, we did not have data on specific extra-glandular manifestations and glandular inflammation. In clinical practice, we usually retain potent immunosuppressive agents, such as azathioprine, cyclophosphamide, methotrexate, or high-dose glucocorticoids to treat severe extra-glandular manifestations or major organ involvement [5, 30, 31]. Therefore, we performed a subgroup analysis stratified by the use of potent immunosuppression and found that pilocarpine may confer protective effects against dental caries in patients who do not take potent immunosuppressive agents, but not in patients who use these drugs. The protective effect of pilocarpine may be present in patients without severe extra-glandular manifestations or without severe inflammation. Therefore, rheumatologists may need to pay more attention to their dental care for patients with severe extra-glandular manifestations because there is no protective effect of pilocarpine in this subgroup.

Patients with pSS have a significantly higher risk of dental caries and dental extractions than the general population throughout their life [2]. According to one study involving patients with pSS, the incidence of caries can be reduced with the use of topical fluoride [32]. Therefore, fluoride might be regarded as the main therapy for preventing dental caries in pSS patients, but it is not supported by any other randomised clinical trial [33]. According to the clinical practice guidelines for oral management in pSS, other preventive strategies, such as salivary stimulants, antimicrobials, and non-fluoride remineralising agents, can be regarded as adjunctive therapy for dental caries [34]. However, studies that specifically involve pSS patients are lacking and should necessarily be carried out to prove the effect of these preventive interventions. Therefore, we should not overestimate the effect of these adjunctive therapies and should suggest the use of topical fluoride although the evidence is also weak.

The limitations of this study include the following. Firstly, there are two salivary stimulants in Taiwan, namely pilocarpine and cevimeline, which are approved by the Taiwanese Food and Drug Administration. Further, pilocarpine and cevimeline are covered by Taiwanese health insurance since 2004 and 2009, respectively. To avoid the possible effects associated with different drug-approval years, as well as the doctor’s choice between these two drugs for uncertain reasons, we excluded patients using cevimeline. Therefore, further studies are necessary to validate the results of the present study, as well as to investigate the effects of other salivary stimulants.

Secondly, some essential defects are present in the national insurance database. For example, data on disease activity and exact salivary flow rate, which may affect the outcomes of clinical trials, are not available in the database. Therefore, besides general variables such as age, sex, and comorbidities, we matched the variables that may be associated with other factors, including immunosuppressant use and healthcare utilisation due to pSS. A considerable effort was made to minimise the impact of this limitation using propensity score matching. Despite this, there may be bias in the analysis due to the lack of data on the patients’ saliva flow rates. Perhaps non-responsiveness is the cause of the insignificant protection. Further research is needed to determine if patients responding to pilocarpine have protection against dental caries.

Finally, although we used a prospective study design, the current study was not a real randomised controlled trial. We did not include the data related to pilocarpine use after the first 3 months of follow-up. Moreover, although health insurance already covers more than 97% of the medical treatment, we were unable to determine whether pSS patients visited the dentist or used topical fluoride at their own expense. Despite these limitations, our research provides the strongest evidence, because no prospective randomised controlled trial concerning the protective effect of pilocarpine against dental caries is available to date.

## Conclusions

In conclusion, pilocarpine therapy seems ineffective for reducing the risk of oral manifestations, such as dental caries, periodontitis, and oral candidiasis, in patients with pSS. Although beneficial for improving salivary flow rate and dry mouth symptoms, the effect of pilocarpine therapy on dental caries prevention is questionable and might be overemphasised. However, observational studies are not the best study design for drug efficacy; thus, a randomised controlled trial is necessary to verify the present findings.

## Supplementary information

**Additional file 1:** **Table S1.** ICD-9-CM code used in the current study.

**Additional file 2: Figure S1.** The effect of pilocarpine on dental caries risk in patients with pSS by medication possession ratio (MPR).

## Data Availability

All data generated or analysed during this study are included in this published article and its supplementary information files. The original databases that support the findings of this study are only used by the National Health Insurance Bureau but cannot be taken out, and therefore are not publicly available.

## Abbreviations

CI: Confidence interval
GEE: Generalised estimating equation
HCQ: Hydroxychloroquine
HR: Hazard ratio
NHIRD: National Health Insurance Research Database
pSS: Primary Sjögren’s syndrome
