# Supplementary material for: The effect of pilocarpine on dental caries in patients with primary Sjögren’s syndrome: a database prospective cohort study
Source: Arthritis Res Ther. 2019 Nov 27;21:251. doi: 10.1186/s13075-019-2031-7 (PMC6882320; doi:10.1186/s13075-019-2031-7)
Supplement: Supplementary file 1 — Additional file 1: Table S1. ICD-9-CM code used in the current study. [file 13075_2019_2031_MOESM1_ESM.docx]

Supplement Table 1 ICD-9-CM code used in the current study

| Variable | Code |
| --- | --- |
| Sjogrene syndrome | 710.2 (Catastrophic Illness Card) |
| Other autoimmune disease | 710.0, 710.1, 714.0, 710.4, 710.3, 443.1, 446.0, 446.2, 446.4, 446.5, 446.7, 446.1, 136.1, 694.4, 555, 556, 556.0, 556.1, 556.2, 556.3, 556.4, 556.5, 556.6, 556.8, 556.9 (Catastrophic Illness Card) |
| Malignancy | 140.xx–208.xx (Catastrophic Illness Card) |
| Hepatitis C virus infection | 070.41, 070.44, 070.51, 070.54, and V02.62 |
| Acquired immunodeficiency syndrome | 042–044, V08 |
| Sarcoidosis | 135 |
| Amyloidosis | 277.3x |
| Organ transplantation | V42.0, V42.1, V42.6, V42.7, V42.8x, 996.81–996.85, |
| Severe asthma | 493.xx with admission |
| Acute iritis | 364.xx |
| Narrow-angle glaucoma | 365.xx |
| **Comorbidity** |  |
| Diabetes mellitus | 250.xx |
| Chronic kidney disease | 580.xx–589.xx, 403.xx–404.xx, 016.0x, 095.4x, 236.9x, 250.4x, 274.1x, 442.1x, 447.3x, 440.1x, 572.4x, 642.1x, 646.2x, 753.1x, 283.11, 403.01, 404.02, 446.21 |
| Cirrhosis | 571.2, 571.5, 571.6 (Catastrophic Illness Card) |
| Chronic obstructive pulmonary disease | 491.xx, 492.xx, 496.xx |
| Stroke | 430.xx–437.xx |
| Dementia | 290.xx, 294.xx with Mini-Mental State Examination (MMSE) |
| **Outcomes** |  |
| Dental caries | 521.0, 521.1, 521.2, 521.3, 522.0, 522.1, 522.2, 522.3, 522.4, 522.5, 522.6, 522.7, 522.8, 522.9 with dental treatment |
| Periodontitis | 523.0, 523.1, 523.2, 523.3, 523.4, 523.5, 523.8, 523.9 with dental treatment |
| Oral candidiasis | 112.0 with antifungal antibiotics |
