# Supplementary figures and images for: The effect of pilocarpine on dental caries in patients with primary Sjögren’s syndrome: a database prospective cohort study
Source: Arthritis Res Ther. 2019 Nov 27;21:251. doi: 10.1186/s13075-019-2031-7 (PMC6882320; doi:10.1186/s13075-019-2031-7)

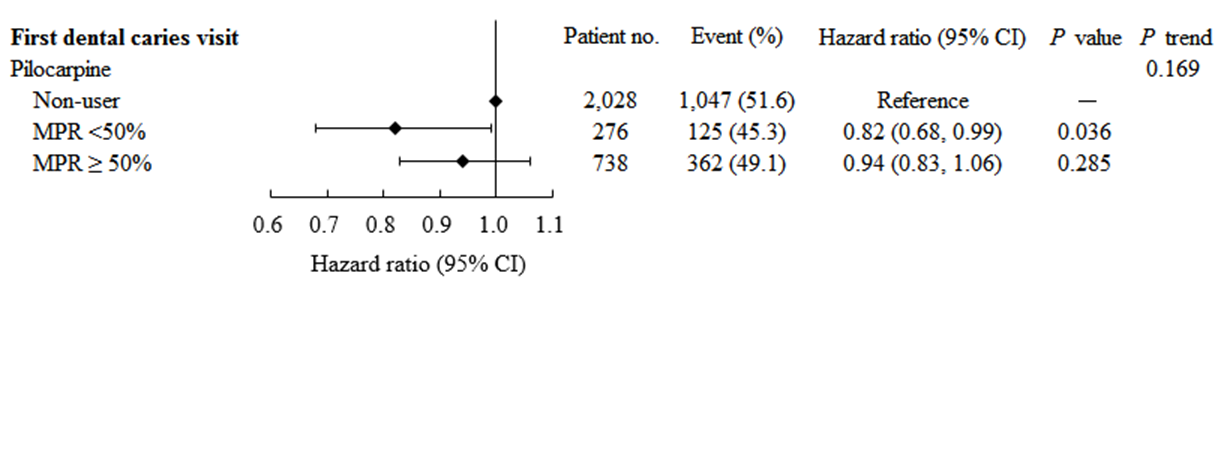

Supplement: Supplementary file 2 — Additional file 2: Figure S1. The effect of pilocarpine on dental caries risk in patients with pSS by medication possession ratio (MPR). [file 13075_2019_2031_MOESM2_ESM.tif]
